# Short- and Long-Term Effects of Conscious, Minimally Conscious and Unconscious Brand Logos

**DOI:** 10.1371/journal.pone.0057738

**Published:** 2013-05-02

**Authors:** Charlotte Muscarella, Gigliola Brintazzoli, Sarah Gordts, Eric Soetens, Eva Van den Bussche

**Affiliations:** Department of Psychology, Vrije Universiteit Brussel, Brussels, Belgium; Cardiff University, United Kingdom

## Abstract

Unconsciously presented information can influence our behavior in an experimental context. However, whether these effects can be translated to a daily life context, such as advertising, is strongly debated. What hampers this translation is the widely accepted notion of the short-livedness of unconscious representations. The effect of unconscious information on behavior is assumed to rapidly vanish within a few hundreds of milliseconds. Using highly familiar brand logos (e.g., the logo of McDonald's) as subliminal and supraliminal primes in two priming experiments, we assessed whether these logos were able to elicit behavioral effects after a short (e.g., 350 ms), a medium (e.g., 1000 ms), and a long (e.g., 5000 ms) interval. Our results demonstrate that when real-life information is presented minimally consciously or even unconsciously, it can influence our subsequent behavior, even when more than five seconds pass between the presentation of the minimally conscious or unconscious information and the behavior on which it exerts its influence.

## Introduction

Ever since James Vicary (fictitiously) claimed in 1957 that he could increase product sales by showing moviegoers unconscious advertisements such as “Drink Coca-Cola” and “Eat popcorn”, this controversial topic has never ceased to stir the public opinion. Nowadays, it is no longer questioned that information, which remains below the consciousness threshold, can influence our behavior in an experimental context [1], [2]. Recently, accumulating evidence indicates that these unconscious influences can even reach very sophisticated levels of cognitive processing [1], [2], [3]. This triggers our curiosity as to whether we can translate these observations to a more daily life context, such as advertising. Several researchers argued that Vicary's claims of unconscious advertising might contain some truth. Experimental goal-priming research focusing on advertising [4], [5], [6], [7], [8] seems to suggest that unconscious primes can influence consumers' choice. For example, Karremans et al. [5] primed participants with the brand name of a drink (i.e., “LIPTON ICE”) and demonstrated that participants' choice and the intention to drink Lipton Ice increased, but only if the participants were thirsty. However, a first major challenge for studies examining unconscious perception is providing sufficient and reliable evidence that the information was indeed presented below the conscious threshold. Abovementioned studies, examining unconscious perception in advertising (e.g., [4], [5], [6], [7], [8]) use subjective report and/or prime identification as a method for assessing prime awareness, which both have been criticized for underestimating prime awareness [see [9] for a more extensive discussion].

Given the apparent difficulties in rendering information completely unconscious and measuring this reliably, especially in a more real-life context (see [4], [5], [6], [7], [8]), the notion of ‘partial awareness’, proposed by Kouider et al. [10], might offer a valuable alternative. According to Kouider et al. [10], different forms of consciousness can be acknowledged, based on a hierarchical view of the levels conscious access. First, *complete awareness* arises when all the levels of representation are accessed, which is associated with a normal state of perception. Second, *complete unawareness* arises when access to any level of representation is absent and when subjects report confidently the absence of a stimulus (e.g., subliminal perception). Finally, *partial awareness* arises when participants are partially aware of a stimulus. More precisely, they have access to a part of the stimulus information (i.e., low-level information) that is transiently activated, but not to all representation levels. This event could arise when the critical stimulus is weak or degraded (e.g., briefly presented, masked). This inaccessible information could be filled up with a perceptual content. More precisely, partially accessible information (bottom-up stimulus related information) would be merged with expectations (top-down information), leading to unreliable accessible information. Thus, beside examining the effects of conscious and (the difficult to achieve and measure) unconscious priming, assessing the potential impact of minimally conscious information on behavior would be a promising approach to study the phenomenon of consciousness. These effects, resulting from a combination of several levels of processing and states inherent at each level, could yield valuable information with regard to the functioning of the different perceptual awareness states and therefrom resulting behavioral effects.

A second problem which hampers whether the conclusions drawn from an experimental context can be translated to an everyday context, is the widely accepted notion of the short-livedness of unconscious representations [11], [12]. Several studies have shown that the effect of unconscious information on behavior rapidly decays and becomes non-significant within a few hundreds of milliseconds [13], [14], [15], [16], [17]. This implies that unconscious information should be able to exert its influence on behavior almost immediately, which casts serious doubt on the applicability of subliminal influences in daily life [18], [19], [20], [21]. However, a few studies did report long-lasting effects of subliminal processing. Capa et al. [22] primed students with subliminal words related to the goal of studying. Afterwards, they participated in an easy or difficult learning task based on their coursework. The authors showed that, on a difficult task, students performed better and had a stronger cardiovascular reactivity related to effort mobilization extending over twenty five minutes. However, this was only observed when the primes had been directly associated with visible positive words acting as a reward. Pessiglione et al. [23] observed a similar finding: they presented subliminal contextual cues paired with monetary outcomes (i.e., −£1, £0, +£1) and asked participants to choose between pressing or not pressing a button, in response to these masked cues. They observed that participants tended to choose cues associated with monetary rewards relative to punishments. At the end of the experiment, participants rated the cues in order of preferences. Ratings were higher for reward compared to punishment cues, indicating a learning of the affective values of the subliminal cues and, thus, long-lasting effects of subliminal processes when linked to visible positive outcomes. Although these studies indicate that subliminal information can have long-term effects on behavior when paired to visible rewards, research on the longevity of unconscious effects remains scarce. Assessing whether and under which circumstances unconscious information can lead to long-term behavioral effects is crucial to determine its potential impact in everyday life.

The aim of our first experiment was to assess the short- and long-term effects of real-life stimuli, which could be presented consciously or minimally consciously. A typical masked priming paradigm [24] was used where brand logo primes (see also [9]) preceded letter string targets, which had to be categorized as words or meaningless pseudo words. This masked priming task was followed by a strict objective prime awareness assessment. In order to assess short- and long-term effects, we manipulated the Stimulus Onset Asynchrony (SOA; i.e., the interval between the onset of the prime and the onset of the target) leading to SOAs of 334 ms, 1000 ms or 5000 ms.

## Experiment 1

### Method

In a priming experiment, participants were asked to perform a lexical decision task on target letter strings. The targets were preceded by masked logo primes. Participants were randomly assigned to two conditions: one group received the conscious condition where primes were presented above the consciousness threshold. The other group received the minimally conscious condition where primes were presented near the consciousness threshold. The time between the onset of the prime and the onset of the target (SOA) was either 334 ms, 1000 ms or 5000 ms, manipulated in three separate blocks of trials.

#### Ethics statement

All procedures were executed in compliance with relevant laws and institutional guidelines. Subjects participated voluntarily or as partial fulfillment of a course requirement. Participants received experimental regulations which stipulated [translated from Dutch]: “Each student is required to read the “Informed Consent” prior to participation. In this Consent, the content of the experiment and the inclusion criteria for the participants are mentioned. In this “Informed Consent” it is always mentioned that you, as a participant, are aware of the content of these regulations and that you agree with them”. Since the data were analyzed completely anonymously (i.e., from the start of the experiment we refrained from registering the participants' names), participants gave oral informed consent before experimentation and signed an attendance list afterwards. They were invited to a debriefing session. The Medical Ethics Committee of the Vrije Universiteit Brussel was consulted and based on our full protocol (including the consent procedure) they decided that our study was exempt from approval (reference 2012/204).

#### Participants

Fifty-five volunteers and psychology students participated in the experiment. Thirty participants were assigned to the conscious condition. Two of them responded significantly (+2*SD*s) slower than the mean and were therefore eliminated from the analyses. Thus, the final sample for the conscious condition consisted of 28 participants (12 men). Their mean age was 22.8 (*SD* = 2.4, range 19–28). Twenty-five participants performed the minimally conscious condition. One subject responded significantly (+2*SD*s) slower than the mean and was therefore eliminated from the analyses. Thus, the final sample for the minimally conscious condition consisted of 24 participants (7 men). Their mean age was 20.2 (*SD* = 1.6, range 18–25).

#### Apparatus

Participants were seated in a dimly lit room, approximately 60 cm from a 15-inch colour CRT monitor connected to a computer running the Windows operating system. Stimulus delivery and the recording of behavioral data (reaction time and accuracy) were controlled by E-prime (www.pstnet.com; Psychology Software Tools).

#### Stimuli

The prime stimuli consisted of five brand logos selected from the stimulus set from Brintazzoli et al. [9]. This stimulus set contained 10 brand logos and from these we selected the 5 logos that elicited the strongest conscious and unconscious priming effects in this previous study (i.e., the logos of McDonald's, Mercedes, Telenet, Nike and Apple, see [9] for the exact stimuli). A pilot study indicated that these logos are highly familiar and easily recognizable for the target population (see [9]). The dimensions of the picture stimuli ranged from 2.9 cm to 3.2 cm in width and 1.4 cm to 3.5 cm in height. The targets, again based on Brintazzoli et al. [9], consisted of 20 words and 20 non-words, ranging from 1.7 cm to 5.4 cm in width and 0.7 cm in height. The word targets could be a brand name or a non-brand Dutch word. The 10 brand targets consisted of the names of logo primes. The 10 non-brand target words were words associated with the logos. The non-brand target words were all medium to high frequency words (log frequencies ranging from 0.30 to 2.32; based on the WordGen program [25]). The non-words were generated using WordGen [25]. Word length was matched in the brand and the non-brand word conditions (on average 7.0 in both conditions). Word length of the target words and the target non-words was also matched (on average 7.0 for both the words and the non-words). Forward and backward masks existed of random dot patterns, 8.5 cm in width and 8.5 cm in height, and constructed such that 4×4 pixels were always chosen randomly to be white or black.

The word targets could either be related or unrelated to the prime. Thus, four target word conditions were created: a) *a related brand condition* where prime and target were related and both described a brand (e.g., McDonald's logo followed by the word “MCDONALD'S”); b) *an unrelated brand condition* where prime and target were not related and both described a brand (e.g., McDonald's logo followed by the word “LACOSTE”); c) *a related non-brand condition* where prime and target were related but the target word was not a brand (e.g., McDonald's logo followed by the word “HAMBURGER”); d) *an unrelated non-brand condition* where prime and target were not related and the target word was not a brand (e.g., McDonald's logo followed by the word “TIRES”). Besides these four target word conditions, a target *non-word condition* was also created, where the logo primes were paired with non-word targets. Each of the five logo primes was paired with a word target from each of the aforementioned four target word conditions. This way, 20 prime-target pairs were created (see Appendix S2). Furthermore, each prime was also paired with four non-words to match the number of word and non-word trials. This led to a total of 40 prime-target pairs.

#### Procedure

**Figure 1:**
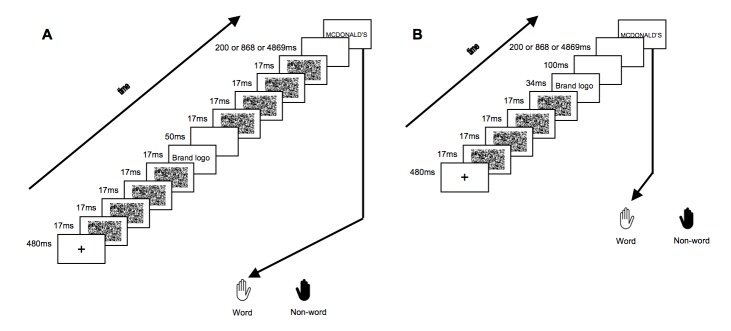
Experimental procedure. A trial started with the presentation of a fixation cross, followed by four random dot masks, and subsequently followed by a brand logo prime. In the minimally conscious version of the experiment (**A**), the logo prime was presented for 17 ms, followed by a blank and four masks. In the conscious version of the experiment (**B**), the logo prime was presented for 34 ms and followed by a blank only. SOAs in the minimally conscious and conscious version were always identical and were determined by the blank preceding the target. This blank could be presented for 200 ms, 868 ms or 4869 ms, leading to SOAs of 334 ms, 1000 ms and 5000 ms respectively.

Figure 1 depicts the sequence of a trial. First, a fixation cross was centrally shown for 480 ms, followed by a forward mask existing of four different random dot patterns with a total duration of 67 ms (i.e., 4×16.7 ms). Following the mask, a picture prime was presented for 17 ms in the minimally conscious condition and for 34 ms in the conscious condition. After the prime, a blank screen was presented for 50 ms. The blank was then followed by a backward mask in the minimally conscious condition (Figure 1A), again existing of four different random dot patterns for 67 ms. In the conscious condition (Figure 1B), these masks were replaced by a blank of the same length. In order to assess short- and long-term effects, we manipulated the SOA by inserting a blank of variable length (i.e., 200 ms, 868 ms or 4869 ms) before the target presentation (see Figure 1). This led to SOAs of 334, 1000 or 5000 ms. Finally, the target was presented until the participants' response was registered. All targets were presented as black capital letters on a white background. A lexical decision paradigm was used: participants were told that they would see letter strings and they were instructed to decide on each trial whether the target letter string was a word or a nonsensical non-word. Participants were not informed about the brand logo primes. Instead they were told that visual flashes would precede these letter strings in order to direct their attention to a central location. When the target was a word they had to press “q”, when it was a non-word they had to press “p” on the keyboard. Participants were instructed to respond as quickly as possible and to avoid mistakes. The inter-trial interval was 1000 ms. All presentations were synchronized with the vertical refresh cycle of the screen (i.e., 16.7 ms). The three SOAs were presented in three different blocks with one SOA per block. The order of these blocks was counterbalanced across participants. In the longest SOA condition, participants received a warning before the block began, indicating that the interval until the stimulus appeared would be long in this block, but asking them to remain focused on the center of the screen.

The 40 prime-target pairs were presented three times leading to 120 experimental trials in each block. The experiment started with 10 practice trials where no prime was shown. Instead of the prime a blank screen was presented. During these practice trials subjects received feedback about their accuracy on the lexical decision task (i.e., the message "correct" or "incorrect"). After these practice trials, the three experimental blocks, each containing 120 experimental trials, were presented. During the experiment, feedback was no longer provided. After each block the participants were able to take a short break.

#### Prime visibility

Prime visibility was assessed using an objective visibility test. After the experiment, participants were informed about the presence of the logo primes and were asked to participate in a post-test in order to assess how well they were able to identify the logo primes. Participants were not informed about the exact nature of the brand logo primes. A prime discrimination task was created, by randomly presenting either the five brand logos used in the priming task, or five grayscale non-brand pictures, with the same dimensions as the brand logos (see Appendix S2). These non-brand picture stimuli were taken from the greyscale shaded images set of the “Snodgrass and Vanderwart-like” objects [26]. The procedure used in this post-test was similar as in the actual experiment with this difference that the participants were asked to categorize the prime: if they thought the prime was a brand logo they had to press "q"; if they thought it was not a brand logo they had to press "p" on the keyboard. If the participants were unable to categorize the primes, they were forced to guess. Contrary to the priming experiment, participants could now take all the time they wanted to categorize the primes in this post-test. Only word targets were presented in this post-test and participants were told to ignore these targets. Twenty of these word trials were identical to the ones in the actual experiment described above (word targets preceded by a brand logo). In addition, the same 20 word targets were preceded by a non-brand picture. This led to a total of 40 trials in this post test, which were presented once. In contrast to the main experiment, only one SOA condition (i.e., 334 ms) was implemented in this prime visibility test.

### Results

Only the trials where the target was a word were included in the analyses. The non-word trials, where no relation is present between the primes and targets, were omitted. Median RTs of correct responses and mean error rates were submitted to a repeated measures analysis with three within-subjects factors: SOA (3 levels: 334 ms, 1000 ms or 5000 ms), prime-target relatedness (2 levels: related or unrelated) and nature of the target (2 levels: brand target or non-brand target) and one between-subjects factor: prime presentation (2 levels: conscious or minimally conscious). Median RTs and mean error rates as a function of these factors are reported in Table 1.

**Table 1:** Means (SD) of the median RTs (in ms) and mean error rates (in%) for the related and unrelated trials and the amount of priming (unrelated - related) as a function of prime presentation (conscious or minimally conscious) and nature of the target (brand target or non-brand target).

			Prime-target relatedness		
Prime presentation	Nature of the target	SOA	Related	Unrelated	Priming
Conscious	Brand	334	482 (56.7)	519 (52.3)	37***
			2.6 (4.6)	8.8 (9.9)	6.2**
		1000	491 (64.2)	527 (57.8)	36***
			1.7 (3.4)	4.5 (6.3)	2.8*
		5000	529 (56.4)	564 (58.9)	35**
			1.9 (3.6)	7.4 (8.8)	5.5**
	Non-Brand	334	469 (60.0)	488 (54.3)	19**
			1.7 (4.7)	3.1 (3.8)	1.4
		1000	476 (44.2)	496 (51.5)	20**
			1.4 (4.2)	3.1 (5.6)	1.7
		5000	523 (68.8)	536 (59.4)	13
			1.7 (3.4)	3.3 (4.6)	1.6
Minimally conscious	Brand	334	492 (32.9)	547 (41.5)	55***
			1.7 (2.9)	6.9 (10.3)	5.2*
		1000	500 (45.2)	539 (46.8)	39***
			2.2 (4.3)	5.6 (8.7)	3.4
		5000	545 (61.4)	571 (63.0)	26**
			2.8 (7.1)	11.9 (9.8)	9.1***
	Non-Brand	334	469 (35.6)	490 (42.1)	21**
			1.9 (5.0)	1.4 (2.8)	−0.5
		1000	496 (44.2)	502 (47.2)	6
			2.2 (3.8)	1.7 (3.5)	−0.5
		5000	538 (68.9)	550 (73.8)	12
			3.3 (8.6)	2.5 (7.0)	−0.8

* *p*<.05; ** *p*<.01; *** *p*<.001.

#### RT analysis

Inaccurate responses (on average 3.6%) were discarded for the RT analyses. The repeated measures analysis revealed a main effect of SOA (*F*(2,49) = 25.08, *p*<.001): subjects responded slower when SOA increased (on average respectively 494.5 ms for an SOA of 334 ms, 503 ms for an SOA of 1000 ms, 544.5 ms for an SOA of 5000 ms). There was a main effect of nature of the target (*F*(1,50) = 65.69, *p*<.001): subjects responded significantly faster on trials where the target was a non-brand (503 ms) as compared to trials where the target was a brand (525.5 ms). The main effect of prime-target relatedness was also significant (*F*(1,50) = 135.27, *p*<.001): subjects responded significantly faster on related trials (501 ms) as compared to unrelated trials (527 ms). Furthermore, the interaction between SOA and nature of the target reached significance (*F*(2,49) = 4.74, *p* = .013): brand targets were always responded to slower than non-brand targets, but this difference was even more prominent for the shorter SOAs. Finally, a significant interaction between nature of the target and prime-target relatedness was observed (*F*(1,50) = 47.07, *p*<.001): the priming effect (i.e., a faster response when prime and target are related) was more prominent in the brand (38 ms) than in the non-brand target condition (15 ms). However, post hoc *t*-tests indicated that both priming effects were significantly different from zero (respectively *t*(51) = 14.45, *p*<.001 and *t*(51) = 5.16, *p*<.001). None of the other effects reached significance.

**Figure 2:**
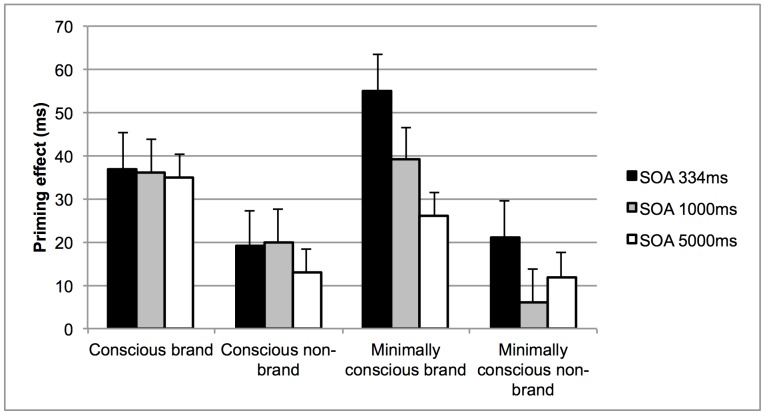
Observed priming effects for Experiment 1. The observed priming effects (i.e., difference in RTs between the unrelated and related trials; in ms) as a function of SOA (i.e., 334 ms, 1000 ms or 5000 ms), nature of the target (i.e., brand or non-brand) and prime presentation (i.e., conscious or minimally conscious). Error bars represent the standard error.

Remarkably, prime-target relatedness did not interact with prime presentation (*F*(1,50) = 0.008, *p* = .93) or SOA (*F*(2,49) = 0.031, *p* = .97) or both (*F*(2,49) = 1.56, *p* = .22), indicating that priming effects were similar in the conscious and minimally conscious condition and across the three SOAs. Post-hoc *t*-tests shed more light on the exact results pattern (see also Table 1). For both the conscious and the minimally conscious condition, significant priming was observed in the *brand target condition*, for all SOAs (for the conscious condition: 37 ms, *t*(27) = 5.48, *p*<.001 for an SOA of 334 ms, 36 ms, *t*(27) = 6.61, *p*<.001 for an SOA of 1000 ms, 35 ms, *t*(27) = 3.86, *p* = .001 for an SOA of 5000 ms; for the minimally conscious: 55 ms, *t*(23) = 8.63, *p*<.001 for an SOA of 334 ms, 39 ms, *t*(23) = 5.24, *p*<.001 for an SOA of 1000 ms, 25 ms, *t*(23) = 3.49, *p* = .002 for an SOA of 5000 ms). For the *non-brand target condition*, significant priming was only observed in the minimally conscious condition for the shortest SOA (21 ms, *t*(23) = 3.82, *p* = .001), but not for the two longer SOAs (both *p*>.22). In the conscious condition, significant priming for the non-brand target condition was found in the 334 ms SOA (19 ms, *t*(27) = 2.81, *p* = .009) and the 1000 ms SOA (20 ms, *t*(27) = 3.52, *p* = .002), but no longer in the 5000 ms SOA (*p* = .12). Figure 2 depicts the observed priming effects as a function of the SOA, nature of the target and prime presentation.

#### Error rate analysis

The same repeated measures analysis was conducted on the mean error rates and revealed a main effect of nature of the target (*F*(1,50) = 28.88, *p*<.001): subjects made significantly more errors on brand (4.8%) as compared to non-brand target trials (2.3%). The main effect of prime-target relatedness was significant (*F*(1,50) = 38.62, *p*<.001): subjects made significantly less mistakes on related trials (2.1%) as compared to unrelated trials (5.0%). The interaction between nature of the target and prime-target relatedness also reached significance (*F*(1,50) = 39.01, *p*<.001): the priming effect (i.e., a more accurate response when prime and target are related) was more prominent in the brand (5.4%) than in the non-brand target condition (0.5%). Post hoc *t*-tests indicated that only the priming effect for the brand target condition was significantly different from zero (*t*(51) = 6.87, *p*<.001). The priming effect for the non-brand target condition did not reach significance (*t*(51) = 1.39, *p* = .17). Finally, a significant three-way interaction between prime presentation, nature of the target and prime-target relatedness was observed (*F*(1,50) = 4.45, *p* = .040): the priming effect was always more prominent for the brand target condition compared to the non-brand target condition, and this difference was stronger in the minimally conscious than the conscious version of the experiment. None of the other effects reached significance. Prime-target relatedness did not interact with SOA, indicating that priming effects were similar across the three SOAs (see also Table 1).

#### Prime visibility

All *p*-values reported in the one-sample *t*-tests are one-tailed. For the *conscious version*, on average, subjects were able to correctly categorize the primes on 94% of the trials. This percentage of prime visibility differed significantly from chance level (*t*(27) = 20.08, *p*<.001). A measure of prime visibility (*d*′) was calculated for each subject. The measures are obtained by treating one level of the response category (i.e., brand logos) as signal and the other level (i.e., non-brand pictures) as noise. The overall mean *d*′ value was 3.25. A *t*-test against the null mean indicated that this *d*′ value was significantly higher than zero (*t*(27) = 17.24, *p*<.001), indicating that the primes were consciously perceived. For the *minimally conscious version*, on average, subjects were able to correctly categorize the primes on 54% of the trials. The percentage of prime visibility differed significantly from chance level (*t*(23) = 1.85, *p* = .039). The overall mean *d*′ value was 0.22, which was significantly different from 0 (*t*(23) = 1.79, *p* = .043), indicating that the primes could be categorized slightly above chance level. For the minimally conscious version, *d*′ was not correlated to the index for the amount of priming for the brand targets (*r* = .14, *F*(1,22) = 0.45, *p* = .51) and the non-brand targets (*r* = −.004, *F*(1,17)<0.001, *p* = .98).

When re-examining the results for the minimally conscious version only for those subjects with a *d*′ value below 1 in this SOA condition (N = 22) or for the median half of the participants with the lowest *d*′ values in this SOA condition (N = 12), the pattern of results remained identical, while prime visibility no longer differed from chance level or was even significantly below chance level (respectively, *t*(21) = 1.05, *p* = .16 and *t*(11) = −1.98, *p* = .036). The only exception was that for the median half of the participants with the lowest *d*′ values, a significant priming effect was also observed in the non-brand target condition at the SOA of 1000 ms (16 ms, *t*(11) = 2.96, *p* = .013).

**Figure 3:**
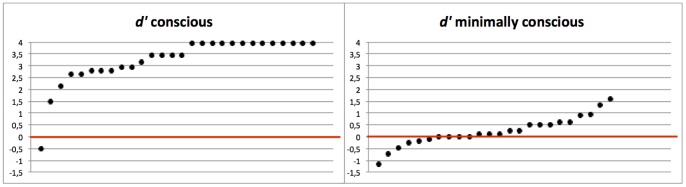
Individual ***d*** **′ values for Experiment 1.** Individual *d*′ values ordered by size (from small to large) for the conscious and the minimally conscious version.

Figure 3 displays the individual *d*′ values ordered by size for the conscious (*d*′ range −1.18–1.56) and the minimally conscious version (*d*′ range −0.51–3.96).

### Discussion

The findings of Experiment 1 indicate that a logo of a familiar brand (e.g., the McDonald's logo) is able to prime its name (e.g., “MCDONALD'S”), even when this logo is presented minimally consciously and when more than 5 seconds pass between the presentation of the minimal conscious information (i.e., the prime) and the behavior on which it exerts its influence (i.e., the categorization of the target). Furthermore, a logo of a familiar brand is also able to prime words strongly related to its brand (e.g., “HAMBURGER”), even when this logo is presented close to the consciousness threshold. However, this process of spreading of activation to semantically related words which seems to be at work here seems to be limited to shorter intervals between prime and target, especially for minimally conscious primes (although for those participants with the lowest prime awareness, significant priming was observed for the SOA of 1000 ms). Thus, the results from Experiment 1 seem to indicate that minimally conscious information can exert its influence on our behavior for at least 5 seconds after its presentation.

However, in our first experiment we did not present the primes entirely below the consciousness threshold. Primes were minimally conscious, but not completely unconscious. Furthermore, one could argue that our post-test was not sufficiently similar to the actual priming experiment. First of all, only one SOA was used in the post-test. Second, the non-brand pictures and the brand logos showed some differences in low-level visual characteristics (e.g., differences in stimulus complexity and stimulus contours). Furthermore, only 40 trials were presented in the post-test. These issues highlight the discussion about the features required in an experimental design in order to warrant reliable conclusion with regards to the unconscious nature of the primes. Although the design of our first experiment met the criteria proposed by Reingold and Merikle [27], Vermeiren and Cleeremans [28] made a critical assessment of *d*′ tasks and although they agree that objective measures are essential when assessing awareness, they stress that the *d*′ task has to be correctly designed for inferences about unconscious processing to be valid. They suggest that neutral targets should be used in the *d*′ task and that a delay should be provided after the prime presentation so that representations of the weak primes can build up and their likelihood of becoming conscious representations is increased. Taking into account these suggestions and by doing so creating a more reliable awareness condition which approaches the consciousness threshold more, we conducted a second experiment. Vermeiren & Cleeremans [28] also suggest to distribute attention in the post-test between the prime and the target. However, since asking participants to solely focus on the prime (instead of distributing attention across prime and target) leads to an overestimation of *d*′ rather than an underestimation and since this would introduce a dual task situation, we did not implement this in our design of Experiment 2. In Experiment 2, a conscious version is no longer used. The primes are presented for a shorter duration and the post-test is thoroughly revised to solve the issues mentioned above: a post-test for each SOA was administered; the visual features of the brand logos and the non-brand pictures were matched more closely; the number of trials was doubled; only neutral targets were presented; and in the two longer SOA conditions, a delay after the prime presentation was automatically present. Using this improved design, we studied whether the results of Experiment 1 reemerged.

## Experiment 2

### Method

#### Ethics statement

The ethics statement mentioned for Experiment 1 also applies for Experiment 2.

#### Participants

Thirty-seven psychology students participated in the experiment. One subject had an average RT above 1000 ms and was therefore eliminated from the analyses. After eliminating this subject, one subject responded significantly (+2*SD*s) slower than the mean and was therefore eliminated from the analyses. One subject was almost perfect in identifying the primes (94% correct prime classifications in the 334 ms SOA condition, 86% in the 1000 ms SOA condition and 84% in the 5000 ms SOA condition). Since we aim to study unconscious processing, this subject was excluded from further analyses. Thus, the final sample consisted of 34 participants (10 men). Their mean age was 19.4 (*SD* = 1.8, range 18–25).

#### Apparatus, stimuli and procedure

Apparatus, stimuli and procedure were highly similar to the minimally conscious version of Experiment 1. However, a few changes were made. Only an unconscious version was presented. To further stimulate the creation of an unconscious version, an 85 Hz (11.7 ms) refresh rate of the screen was used, which slightly changed the timing of the events. First, a fixation cross was centrally shown for 480 ms, followed by a forward mask existing of four different random dot patterns with a total duration of 48 ms (i.e., 4×12 ms). Following the mask, a picture prime was presented for 12 ms. After the prime, a blank screen was presented for 24 ms. The blank was then followed by a backward mask, again existing of four different random dot patterns for 48 ms. In order to assess short- and long-term effects, we manipulated the SOA by inserting a blank of variable length (i.e., 199 ms, 854 ms or 4867 ms) before the target presentation (see Figure 1). This led to SOAs of 284, 939 or 4952 ms. Finally, the target was presented until the participants' response was registered.

Response assignment was now randomly varied across participants: 18 of the participants pressed “q” when the target was a word and “p” when it was a non-word, whereas 16 participants performed the experiment with the opposite response assignment.

Prime visibility was again assessed using an objective visibility test. A prime discrimination task was created, by randomly presenting either five grayscale non-brand pictures, or the five brand logos. The non-brand pictures were different from Experiment 1, to better match the complexity, greyscale colors and dimensions of the logos (see Appendix S3). These non-brand pictures were taken from “Clker.com” (http://www.clker.com), an online sharing service where users share free public domain vector cliparts, except for the airplane picture. However, Appendix S3 shows a highly similar picture from an airplane taken from “Clker.com”. The color and size of all non-brand pictures was adapted to perfectly match the brand logos. Only non-word target trials were presented in this post-test (see the suggestion of Vermeiren & Cleeremans [28]). The 40 trials in this post-test were presented twice instead of once in Experiment 1. Similar to the priming experiment, three SOA conditions (i.e., 284, 939 and 4952 ms) were implemented in the prime visibility post-test. The order of these three SOA blocks in the post-test was identical to the order the participant received in the priming experiment.

The rest of the procedure and stimuli were identical to Experiment 1.

### Results

Only the trials where the target was a word were included in the analyses. The non-word trials, where no relation was present between the primes and targets, were omitted. Median RTs of correct responses and mean error rates were submitted to a repeated measures analysis with three within-subjects factors: SOA (3 levels: 284 ms, 939 or 4952 ms), prime-target relatedness (2 levels: related or unrelated) and nature of the target (2 levels: brand target or non-brand target). Median RTs and mean error rates as a function of these factors are reported in Table 2.

**Table 2:** Means (SD) of the median RTs (in ms) and mean error rates (in%) for the related and unrelated trials and the amount of priming (unrelated - related) as a function of nature of the target (brand target or non-brand target).

		Prime-target relatedness		
Nature of the target	SOA	Related	Unrelated	Priming
Brand	284	539 (63.0)	584 (71.0)	45***
		3.1 (5.0)	8.2 (11.6)	5.1*
	939	550 (56.7)	599 (71.9)	49***
		2.3 (4.0)	5.5 (8.1)	3.2
	4952	601 (86.1)	661 (125.2)	60***
		2.3 (4.3)	7.1 (8.5)	4.8**
Non-Brand	284	533 (69.3)	537 (71.0)	4
		2.3 (7.3)	2.0 (3.9)	−0.3
	939	540 (54.7)	553 (54.0)	13*
		0.4 (1.6)	1.8 (5.3)	1.4
	4952	590 (82.5)	610 (105.0)	20*
		1.8 (3.4)	1.8 (4.1)	0

* *p*<.05; ** *p*<.01; *** *p* <.001.

#### RT analysis

Inaccurate responses (on average 3.2%) were discarded for the RT analyses. The repeated measures analysis revealed a main effect of SOA (*F*(2,32) = 11.54, *p*<.001): subjects responded slower when SOA increased (on average respectively 548 ms for an SOA of 284 ms, 560 ms for an SOA of 939 ms, 615 ms for an SOA of 4952 ms). There was a main effect of nature of the target (*F*(1,33) = 29.85, *p*<.001): subjects responded significantly faster on trials where the target was a non-brand (560 ms) as compared to trials where the target was a brand (589 ms). The main effect of prime-target relatedness was also significant (*F*(1,33) = 53.98, *p*<.001): subjects responded significantly faster on related trials (559 ms) as compared to unrelated trials (591 ms). Furthermore, a significant interaction between nature of the target and prime-target relatedness was observed (*F*(1,33) = 32.13, *p*<.001): the priming effect (i.e., a faster response when prime and target are related) was more prominent in the brand (52 ms) than in the non-brand target condition (12 ms). However, post hoc *t*-tests indicated that both priming effects were significantly different from zero (respectively *t*(33) = 7.65, *p*<.001 and *t*(33) = 3.07, *p* = .004). None of the other effects reached significance.

**Figure 4:**
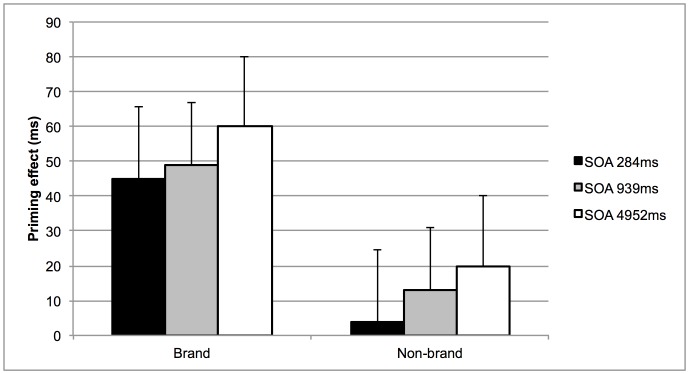
Observed priming effects in Experiment 2. The observed priming effects (i.e., difference in RTs between the unrelated and related trials; in ms) as a function of SOA (i.e., 334 ms, 1000 ms or 5000 ms) and nature of the target (i.e., brand or non-brand). Error bars represent the standard error.

Remarkably, prime-target relatedness did not interact with SOA (*F*(2,33) = 1.11, *p* = .34), indicating that priming effects were similar across the three SOAs. Post-hoc *t*-tests shed more light on the exact results pattern (see also Table 2). Significant priming was observed in the *brand target condition*, for all SOAs (45 ms, *t*(33) = 6.26, *p*<.001 for an SOA of 284 ms, 49 ms, *t*(33) = 6.27, *p*<.001 for an SOA of 939 ms, 60 ms, *t*(33) = 4.06, *p*<.001 for an SOA of 4952 ms). For the *non-brand target condition*, significant priming was not observed for the shortest SOA (3 ms, *F*<1), but for the two longer SOAs significant priming did emerge (13 ms, *t*(33) = 2.34, *p* = .025 for an SOA of 939 ms; 21 ms, *t*(33) = 2.09, *p* = .044 for an SOA of 4952 ms). Figure 4 depicts the observed priming effects as a function of the SOA and nature of the target.

#### Error rate analysis

The same repeated measures analysis was conducted on the mean error rates and revealed a main effect of nature of the target (*F*(1,33) = 17.26, *p*<.001): subjects made significantly more errors on brand (4.7%) as compared to non-brand target trials (1.7%). The main effect of prime-target relatedness was significant (*F*(1,33) = 10.90, *p* = .002): subjects made significantly less mistakes on related trials (2.0%) as compared to unrelated trials (4.4%). The interaction between nature of the target and prime-target relatedness also reached significance (*F*(1,33) = 6.64, *p* = .015): the priming effect (i.e., a more accurate response when prime and target are related) was more prominent in the brand (4.3%) than in the non-brand target condition (0.3%). Post hoc *t*-tests indicated that only the priming effect for the brand target condition was significantly different from zero (*t*(33) = 2.99, *p* = .005). The priming effect for the non-brand target condition did not reach significance (*t*(33) = 1.04, *p* = .30).

None of the other effects reached significance. Prime-target relatedness did not interact with SOA, indicating that priming effects were similar across the three SOAs (see also Table 2).

#### Prime visibility

All *p*-values reported in the one-sample *t*-tests are one-tailed. Subjects were able to correctly categorize the primes on 52% of the trials for an SOA of 284 ms (*t*(33) = 1.10, *p* = .14), 54% for an SOA of 939 ms (*t*(33) = 2.22, *p* = .016) and 51% for an SOA of 4952 ms (*t*(33) = 0.67, *p* = .25). The overall mean *d*′ values were 0.14 for an SOA of 284 ms (*t*(33) = 1.11, *p* = .14), 0.19 for an SOA of 939 ms (*t*(33) = 1.74, *p* = .046) and 0.10 for an SOA of 4952 ms (*t*(33) = 0.66, *p* = .26). Thus, in the 939 ms SOA condition, primes could be categorized slightly above chance level, indicating that the primes in this condition were minimally conscious. When re-examining the results for the 939 ms SOA condition only for those subjects with a *d*′ value below 1 in this SOA condition (N = 31) or for the median half of the participants with the lowest *d*′ values in this SOA condition (N = 17), the pattern of results remained identical, while prime visibility no longer differed from chance level or was even significantly below chance level (respectively, *t*(30) = 0.07, *p* = .23 and *t*(16) = −2.85, *p* = .012). In the other two SOA conditions primes could not be categorized above chance level.

**Figure 5:**
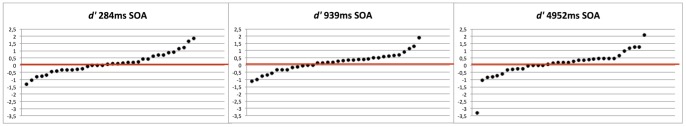
Individual ***d*** **′ values for Experiment 2.** Individual *d*′ values ordered by size (from small to large).

In all SOA conditions, *d*′ was never correlated to the index for the amount of priming for the brand targets (all *p*>.26) and the non-brand targets (all *p*>.18). Figure 5 displays the individual *d*′ values ordered by size for the SOA of 284 ms (*d*′ range −1.32–1.83), the SOA of 939 ms (*d*′ range −1.12–1.90) and the SOA of 4952 ms (*d*′ range −3.29–2.09) separately.

## General Discussion

The aim of this study was to investigate whether the well-established unconscious effects on behavior in an experimental context have the potential to be translated to a more applied context, such as advertising. A key premise for unconscious information influencing our behavior in daily life, is its potential to exert *long-term* effects on behavior, which is subject to great doubt. In order to address this question, we assessed whether conscious, minimally conscious or unconscious real-life stimuli can have short- and long-term effects on our behavior. Two priming experiments were used where primes were highly familiar brand logos (e.g., McDonald's logo) which could be either consciously, minimally consciously Experiment 1) or unconsciously (Experiment 2) presented to participants. SOA was manipulated to be able to study the short- and long-term effects.

The current results indicate that a logo of a familiar brand (e.g., the McDonald's logo) is able to prime its name (e.g., “MCDONALD'S”), even when this logo is presented minimally consciously or even unconsciously and when more than 5 seconds pass between the presentation of the unconscious information (i.e., the prime) and the behavior on which it exerts its influence (i.e., the categorization of the target). Furthermore, a logo of a highly familiar brand is also able to prime words strongly related to its brand (e.g., “HAMBURGER”), even when this logo is presented minimally consciously or unconsciously. However, this process of spreading of activation to semantically related words which seems to be at work here shows a rather capricious pattern: in Experiment 1 it is only found for the shortest SOA for the minimally conscious primes (and for the two shortest SOAs for the half of the participants with the lowest *d*′ values) and for the two shortest SOAs for the fully conscious primes. In Experiment 2, we also observe unconscious priming effects for the non-brand condition after the 1 and 5 second SOAs, but not after the shortest SOA. These discrepancies could be due to the difference between the conscious, minimally conscious and unconscious conditions and thus indicative of qualitative differences between different consciousness states. However, it seems equally plausible that this merely indicates that the effects for non-brand targets are unstable (without a doubt less stable than the effect for brand targets) and require more power to reach a stronger level. In any case, the spreading of activation of logo primes to associated words requires further investigation.

Our finding that unconscious information can exert its influence on our behavior for at least 5 seconds after its presentation is in line with some previous studies [22], [23], but we also demonstrated that a direct association between the unconscious information and a clearly visible positive valence is not a necessary condition to obtain long-lasting unconscious effects (although the brand logos might hold a positive valence for people, this valence was not clearly visible in the minimally conscious and unconscious conditions). Why do we observe robust long-term effects of unconscious information, while so many others have failed to establish this? The nature of the unconscious information seems to be the crucial factor. Highly familiar brand logos prove to be very powerful primes: in comparison to other, non-brand pictures [29], they trigger very robust and numerically large priming effects (see Tables 1 and 2 and [9]). Apparently, this kind of logos have formed very strong representations which, when presented unconsciously, are not short-lived. We stress however, that not all brand logos show this property: when adding more logos to the paradigm, which are slightly less familiar and well-known, no unconscious priming effects whatsoever were observed [9].

While in Experiment 1 primes were presented minimally consciously, we aimed to create reliable unconscious conditions in Experiment 2. In the shortest and longest SOA conditions primes were indeed presented below the consciousness threshold. However, for the SOA of 939 ms, we still observed above chance correct prime categorization. This is in line with the suggestion of Vermeiren and Cleeremans [28], who demonstrated that inserting a delay after the prime leads to increased *d*′ values. Furthermore, one could argue that in the 4952 ms SOA condition the delay was too long for the representation of the prime to last and thus to become aware. However, this is contradicted by the observation of significant and strong priming effects in the 4952 ms SOA condition, which indicates that the prime's representation had not decayed completely. Furthermore, when studying the results excluding subjects with above chance prime visibility in the 939 ms SOA condition, the pattern of results remained completely identical. Thus, in Experiment 2 we used a design to optimize *d*′ assessment, but we still observed significant short- and long-term priming in the absence of prime visibility.

In this experimental study robust long-term effects (i.e., over a five second interval) of minimally conscious and unconscious information were observed in an artificial laboratory context. We stress however that in an everyday context, where competing stimuli are prominently present, priming effects need to be much stronger in order to overcome this competition. Furthermore, priming effects lasting over longer intervals are essential so that the larger time-gap between the presentation of the unconscious information and the subsequent influence on behavior that is characteristic for an everyday context can be bridged. Still, determining that minimally conscious and unconscious real-life stimuli, related to an advertising context, can have long-term effects on our behavior in an experimental context is a first step in assessing their potential impact in everyday life. Although we studied the influence of highly familiar brand logos on low-level categorization behavior, these stimuli prove to be ideal candidates to be used to investigate their impact on consumer behavior and decision making as well.

## Supporting Information

Appendix S1 **Prime-target pairs used in the experiment (and their English translations if different from the Dutch word). Each of the five brand logo primes was paired with four word targets (a related brand target, an unrelated brand target, a related non-brand target, an unrelated non-brand target and four pseudoword targets.**
(DOC)

Appendix S2 **Non-brand picture primes used in the prime visibility post-test of Experiment 1 and their names.**
(DOC)

Appendix S3 **Non-brand picture primes used in the prime visibility post-test of Experiment 2 and their names.**
(DOC)
