# Supplementary material for: Short- and Long-Term Effects of Conscious, Minimally Conscious and Unconscious Brand Logos
Source: PLoS One. 2013 May 2;8(5):e57738. doi: 10.1371/journal.pone.0057738 (PMC3642191; doi:10.1371/journal.pone.0057738)
Supplement: Appendix S1 — Prime-target pairs used in the experiment (and their English translations if different from the Dutch word). Each of the five brand logo primes was paired with four word targets (a related brand target, an unrelated brand target, a related non-brand target, an unrelated non-brand target and four pseudoword targets. (DOC) [file pone.0057738.s001.doc]

APPENDIX S1. Prime-target pairs used in the experiment (and their English translations if different from the Dutch word). Each of the five brand logo primes was paired with four word targets (a related brand target, an unrelated brand target, a related non-brand target, an unrelated non-brand target- and four pseudoword targets.

| **Logo prime** | **Related brand target** | **Unrelated brand target** | **Related non-brand target** | **Unrelated non-brand target** |
| --- | --- | --- | --- | --- |
| Mercedes | Mercedes | Côte d'or | Auto (car) | KLEDING (clothes) |
| Apple | Apple | Michelin | Computer | NAAKT (nudity) |
| Nike | Nike | DELHAIZE | Sport (sports) | chocolade (chocolate) |
| McDonald's | McDonald's | Lacoste | Hamburger | BANDEN (tires) |
| Telenet | Telenet | PLAYBOY | INTERNET | supermarkt (supermarket) |
