# Supplementary material for: Short- and Long-Term Effects of Conscious, Minimally Conscious and Unconscious Brand Logos
Source: PLoS One. 2013 May 2;8(5):e57738. doi: 10.1371/journal.pone.0057738 (PMC3642191; doi:10.1371/journal.pone.0057738)
Supplement: Appendix S2 — Non-brand picture primes used in the prime visibility post-test of Experiment 1 and their names. (DOC) [file pone.0057738.s002.doc]

**APPENDIX S2. Non-brand picture primes used in the prime visibility post-test of Experiment 1 and their names.**

| **Non-brand picture** | **Name** |
| --- | --- |
| 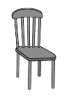 | CHAIR |
| 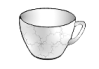 | CUP |
| 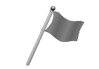 | FLAG |
| 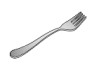 | FORK |
| 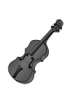 | VIOLIN |
