# Supplementary material for: Short- and Long-Term Effects of Conscious, Minimally Conscious and Unconscious Brand Logos
Source: PLoS One. 2013 May 2;8(5):e57738. doi: 10.1371/journal.pone.0057738 (PMC3642191; doi:10.1371/journal.pone.0057738)
Supplement: Appendix S3 — Non-brand picture primes used in the prime visibility post-test of Experiment 2 and their names. (DOC) [file pone.0057738.s003.doc]

**APPENDIX S3. Non-brand picture primes used in the prime visibility post-test of Experiment 2 and their names.**

| **Non-brand picture** | **Name** |
| --- | --- |
| 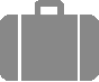 | SUITCASE |
| 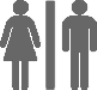 | TOILET |
| 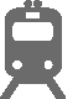 | TRAIN |
| 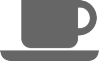 | COFFEE |
| 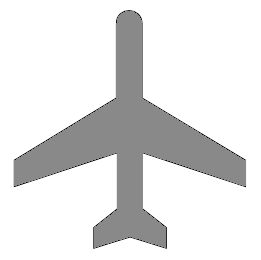 | AIRPLANE |

*Note*. The airplane symbol depicted in Appendix S3 (taken from “Clkr.com”) is slightly different from the one used in the actual Experiment. More specifically, the airplane picture included in the experiment was tilted 45° to the right, was less sharp-edged and included a rudder). 
